# An Intuitive Approach to the Optimal Sampling of an Electromagnetic Field

**DOI:** 10.3390/s25247591

**Published:** 2025-12-14

**Authors:** Marco Donald Migliore

**Affiliations:** 1 Dipartimento di Ingegneria Elettrica e dell’Informazione “Maurizio Scarano” (DIEI), University of Cassino and Southern Lazio, via G. Di Biasio 43, 03043 Cassino, Italy; mdmiglio@unicas.it; 2 European University of Technology, European Union, Cassino, Italy, 03043 Cassino, Italy

**Keywords:** non-redundant sampling, antennas, measurement

## Abstract

The goal of this article is to provide an intuitive explanation of an electromagnetic field sampling technique, known as optimal field sampling. This explanation provides insight into the physical mechanisms underlying the sampling strategy along observation curves.

## 1. Introduction

The goal of this paper is to provide an intuitive explanation of an important theory in the field of high-frequency applied electromagnetics, known as optimal sampling (or minimum redundancy sampling) of an electromagnetic field [1,2,3]. The discussion is restricted to observation curves, while the case of observation planes is only briefly outlined.

The theory was initially developed in the context of antennas, with particular reference to near-field measurements. A key practical challenge in near-field measurements is to minimize the number of spatial samples required to achieve a target accuracy [4]. Reducing the sample count can translate directly into shorter test times, lower operating costs, and higher throughput, without compromising measurement fidelity. In this context, the optimal sampling strategies introduced in [1,2,3] offer a rigorous and effective solution [5]. These methods rely on a robust mathematical framework to identify where, and at what density, to sample in order to meet a prescribed accuracy with the fewest possible measurements. This field of application has been subject of a large literature, with a large number of scientific papers, extensively reported in [6].

However, the efficiency and effectiveness of the optimal sampling quickly extended its application far beyond antenna measurement, and nowadays applications of the optimal sampling theory cover almost all high-frequency electromagnetic disciplines. A review of the applications of the theory is reported in [7].

Before entering into the details of the theory, it is essential to clarify what is meant by optimality of the sampling technique.

Loosely speaking, an electromagnetic problem can be recast as a process that goes from some a priori information on the radiating system to a posteriori information that reduces the set of possible solutions, ideally to a single element of the search space. The number of measurements represents the information gain and depends on the initial set, i.e., on the available a priori information. Adding more a priori information reduces the search space, thereby requiring less “information gain”. The main challenge is to identify suitable and reliable a priori information about the electromagnetic problem and to develop effective algorithms capable of exploiting such information.

The procedure proposed in [1,3] exploits simple, readily available a priori information about the source (e.g., position, shape) and the observation domain (e.g., shape, regularity characteristics) to shrink the feasible set of configurations, and a fast and highly efficient interpolation method based on Shannon–Whittaker–Kotelnikov series, using sinc-function bases. The method’s strength lies in the practicality of these assumptions—which makes the approach easy to deploy—in their effectiveness at reducing the number of required measurements to a value close to the practical minimum, and in the highly efficient interpolation formula. It is understood, however, that different a priori models induce different search spaces and may permit even fewer measurements; for example, in sparse-source settings the measurement burden can drop by orders of magnitude [8]. This method exploits the sparsity of the source as a priori information, thereby drastically reducing the solution search space, but with a higher computational cost, requiring a convex minimization.

As previously noted, this paper presents an intuitive, practice-oriented explanation of the principle at the basis of this sampling technique. This explanation, rather than dwelling on full mathematical details, emphasizes a physical interpretation of the underlying theory. Section 2 of the paper discusses the theoretical lower bound on the number of measurements in field representation by linear interpolation using a functional approach grounded in the results of [2]. Section 3 presents the sampling representation of the field along a curve and summarizes the main results of optimal sampling theory without delving into mathematical details, for which interested readers are referred to [1,2,3]. Section 4 develops an intuitive approach to sampling on curves. Some general indications regarding possible extensions to 2D surfaces are reported. Finally, Section 5 concludes the paper.

## 2. Estimation of the Lower Bound of the Number of Samples in NF Measurements

The aim of this Section is to identify a lower bound for the number of measurements required to represent the field using linear representations of a source whose geometry and position in the space is known.

**Figure 1:**
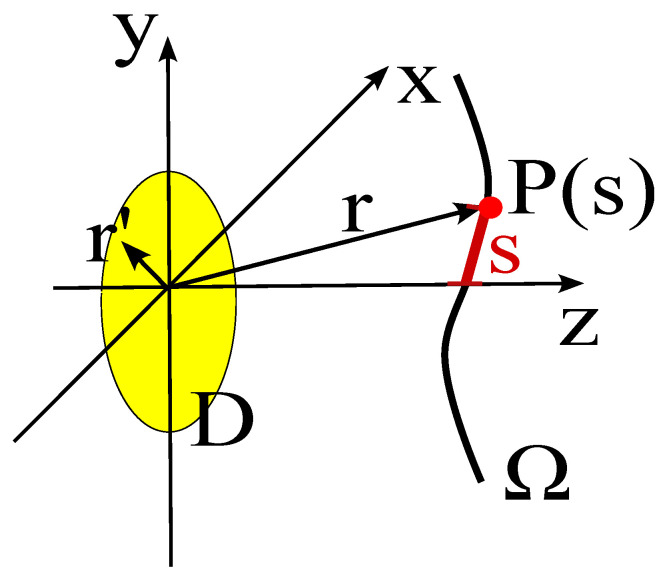
Geometry of the problem: *D* is the source domain, Ω is the observation curve, *s* is the curvilinear abscissa, $r^{\prime }$ denotes the position vector of a source point within *D*, and r denotes the position vector of an observation point on Ω.

In order to identify the optimal representation in the mean–square–error sense (i.e., in the $L_{2}$ norm), let us model the antenna under test (AUT) as a time-harmonic electromagnetic source placed in a bounded domain *D* (see Figure 1). The field, observed on the domain Ω (assumed not to intersect *D*), is given by [1].

(1) $$E\left(r\right)\;=\;\int_{D}\bar{G}\left(r,r^{\prime }\right)\cdot J\left(r^{\prime }\right)\;dr^{\prime },\;r\in \Omega ,$$

where $J(r^{\prime })$ is the source current density, $\bar{G}\left(r,r^{\prime }\right)$ is the free-space dyadic Green’s function [4], and “·” denotes the dyadic–vector product.

In the following discussion we suppose that the currents and the fields belong to separable Hilbert spaces equipped with the $L_{2}$ norm. The set of admissible currents is supposed to be bounded with unit radius.

The radiation operator can be diagonalized using the Hilbert–Schmidt expansion [9,10]. In particular, there exist non-negative singular values ${\left\{\sigma_{n}\right\}}_{n\geq 1}$, an orthonormal set of left singular functions $\left\{u_{n}\right\}$, and an orthonormal set of right singular functions $\left\{v_{n}\right\}$ such that

(2) $$E\left(r\right)=\sum_{n=1}^{\infty }\sigma_{n}\;\langle J_{n}\left(r^{\prime }\right),v\left(r^{\prime }\right)\rangle \;u_{n}\left(r\right),$$

where $\sigma_{1}\geq \sigma_{2}\geq \cdots \geq \sigma_{n}\geq \cdots \to 0$ and 〈·,·〉 denotes the $L_{2}\left(D\right)$ inner product.

A fundamental result of approximation theory states that truncating the series to $\overset{¯}{N}$ terms, with $\overset{¯}{N}$ equal to the number of singular values above ϵ plus one, guarantees an approximation error not exceeding ε. This number $\overset{¯}{N}$ is called the Number of Degrees of Freedom of the field at ε level of approximation, ${\mathrm{NDF}}_{\varepsilon }$:

(3) $$E\left(r\right)≃\sum_{n=1}^{{\mathrm{NDF}}_{\varepsilon }}\sigma_{n}\;\langle J_{n}\left(r^{\prime }\right),v\left(r^{\prime }\right)\rangle \;u_{n}\left(r\right).$$

This number is also the minimum number of measurements required to represent the field within the required approximation using linear interpolation functionals. Summarizing, the Hilbert–Schmidt decomposition allows one to obtain both the optimal basis functions to represent the field on the measurement domain Ω (i.e., the left singular functions) and the number of basis functions required to represent the field within a given approximation, which equals the number of singular values greater than ϵ.

In the asymptotic regime (βa→∞), where β is the free-space wavenumber and *a* is a characteristic dimension of the radiating structure (e.g., its maximum length in the case of a linear source, or the radius in the case of a sphere), the decay of the singular values beyond the knee becomes extremely fast, and the number of terms in the series becomes essentially insensitive to the choice of ε. This allows one to introduce the concept of the Number of Degrees of Freedom (NDF) independently of the specific value of ε [2].

In practical applications, the parameter ε is selected so that, with very high probability, the random noise affecting the measurements does not exceed ε. In general, throughout the paper we will assume that the asymptotic regime has been reached and we will use NDF accordingly, resorting to ${\mathrm{NDF}}_{\varepsilon }$ only when strictly necessary.

It must be also noted that in real scenarios the effective number of basis functions ${\mathrm{NDF}}_{\varepsilon }$ required to approximate the field with accuracy ε in the Fresnel or Fraunhofer region is typically only slightly larger than the asymptotic number of degrees of freedom NDF [1]. Analytical upper bounds on the representation error are provided in [2].

In the following discussion, we analyze a single component of the current and the corresponding component of the electric field, thereby reducing the vector problem to a scalar one. This simplifies the mathematical expressions without losing any conceptually important detail.

## 3. Sampling the Field

In this section, the discussion will be focused on an observation curve, on which we have defined a curvilinear abscissa *s*. Consequently, the points on the observation curve are defined by the position vector r(s) (see Figure 1). For the sake of notational simplicity, we will often indicate the position of the observation point simply with its abscissa *s*. Furthermore, in all the subsequent discussion, we will assume that the observation curve lies outside the reactive zone of the radiating system.

Representation (3) allows one to interpolate the field using ${\mathrm{NDF}}_{\varepsilon }$ parameters. However, such parameters cannot be directly measured at single points in space, since they require computing the inner product between the field on the domain of observation and the singular functions.

A more useful series from an application point of view is the Shannon–Whittaker–Kotelnikov series, using sinc-function bases. Projecting the field onto the Paley–Wiener space, we obtain the $E_{w^{\prime }}\left(s\right)$ band-limited representation of the field:

(4) $$\begin{array}{cc} E_{w^{\prime }}\left(s\right)\; & =\;\sum_{k\in Z}E\left(s_{k}\right)\;\mathrm{sinc}\;\left(w^{\prime }\left(s-s_{k}\right)\right) \end{array}$$

where sinc(x)=sin(x)/x, $s_{k}=k\;\Delta s$, $\Delta s\;=\;\frac{\pi }{w^{\prime }}$, and $w^{\prime }$ is the spatial bandwidth.

For practical applications, it is required to truncate the series to, for example, $N^{\prime }$ terms. However the field on the observation curve is not a band-limited function. This does not prevent representing the field within any prescribed accuracy—since the set of band-limited functions is dense in the set of fields on the observation curve—but it can greatly increase the number of basis functions required, often yielding a largely redundant representation.

To reduce the redundancy of the series, instead of the field itself, the authors of [1,2,3] introduced an optimal sampling strategy, that takes advantage of some a priori information on the position and size of the radiating source to decrease the number of samples required to interpolate the field. Loosely speaking, the idea is to change the function to be projected onto the Paley–Wiener space. Instead of E(s), a “reduced” field is considered, obtained by extracting a suitable phase function Ψ(ξ) and introducing a suitable parameterization ξ(s) of the observation curve:

(5) $$F\left(\xi \right)\;=\;E\left(\xi \right)\;e^{j\Psi (\xi )}.$$

This mapping is used to embed geometrical information into the sampling process.

The corresponding sampling series is

(6) $$F_{w}\left(\xi \right)\;=\;\sum_{k=-N}^{N}F\left(\xi_{k}\right)\;\mathrm{sinc}\;\left(w(\xi -\xi_{k})\right),$$

where $\Delta \xi \;=\;\frac{\pi }{w}$ and $\xi_{k}=k\;\Delta \xi$.

With a suitable choice of the functions Ψ(s) and ξ(s), the quantity $F_{w}$ can be represented with far fewer samples than (6) for a given maximum approximation error. Since reconstructing the field by inverting (5) clearly requires the same number of samples as $F_{w}$, representation of the field using (4) is more efficient than one obtained directly from field samples.

The functions ξ(s) and Ψ(s), which depend on the source and observation geometries, are obtained via an optimization procedure [3]. This procedure determines the function Ψ(ξ) that minimizes the field’s local bandwidth—i.e., the bandwidth over a short segment around each observation point *s*. It also finds the optimal parameterization ξ(s) that keeps constant the local bandwidth, thereby avoiding unnecessary oversampling along portions of the curve. In short, instead of relying on the field’s global bandwidth, the method exploits local spatial spectral properties to achieve an accurate representation with the minimum total number of samples.

**Figure 2:**
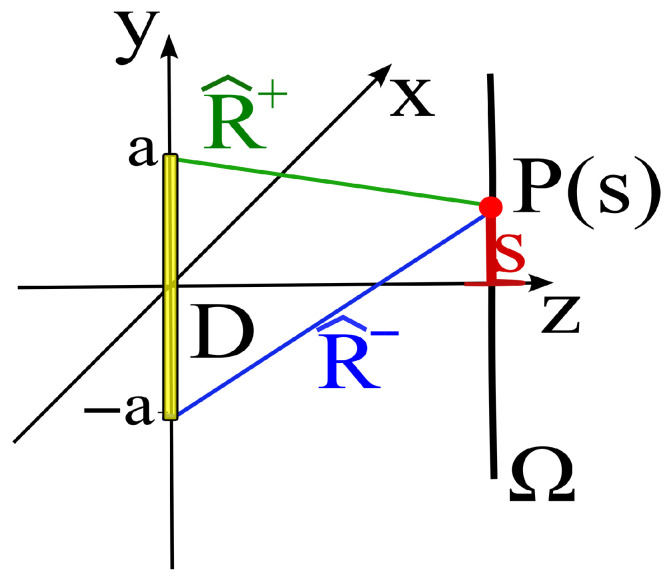
Geometry of the problem: *D* is a linear source of length ℓ=2a, Ω is a linear observation curve, and *s* denotes the rectilinear abscissa.

The theory provides analytical expressions for the main source geometries, which are very useful in practical applications. For example, with reference to the practically relevant case of a linear source and a linear observation curve, the functions are explicitly reported in [11] (see Figure 2), giving

(7) $$\begin{array}{cc} \Psi (s) & =\beta \;\frac{{\hat{R}}^{+}\left(s\right)+{\hat{R}}^{-}\left(s\right)}{2}, \end{array}$$

(8) $$\begin{array}{cc} \xi (s) & =\frac{{\hat{R}}^{+}\left(s\right)-{\hat{R}}^{-}\left(s\right)}{2a}, \end{array}$$

(9) $$\begin{array}{cc} W & =\beta a, \end{array}$$

where ${\hat{R}}^{+}\left(s\right)$ and ${\hat{R}}^{-}\left(s\right)$ denote the distances between the so-called extremal points of the source and the observation point P(s). In general, the extremal points are the points on the source surface where the lines originating from P(s) are tangent to the surface.

In the present example, involving a linear source, they reduce to the endpoints of the segment, i.e., the points (0,±a). The mathematical details are reported in [3].

In [1] it is demonstrated that it is possible to approximate *F* with a band-limited function within any degree of accuracy by considering a slightly larger bandwidth compared to a value *W* called the effective bandwith, say w=χW, where χ>1 is a band limitation enlargement factor. The approximation error between *F* and its w=χW band limited version rapidly tends to zero as χ increases. A value of χ around 1.2 is generally sufficient to ensure that the band limitation error is well below the noise level affecting the measured data. Analytic upper bounds on the representation error are available [2], which reinforce the rigorous theoretical foundation of the optimal sampling representation. Experimental results reported in [11] fully confirm the results of the theory.

In summary, the above theory enables a simple and effective representation of the electromagnetic field using a number of field samples *N* that is only slightly greater than the minimum number of samples required by any linear representation of the field, namely ${\mathrm{NDF}}_{\varepsilon }$.

## 4. An Intuitive Physical Explanation of the Optimal Sampling Method

As a preliminary step, let us consider the classic Shannon–Whittaker–Kotelnikov series representation of a bandlimited signal in the time domain:

(10) $$\begin{array}{cc} x(t) & =\sum_{n\in Z}x\left(t_{n}\right)\;\mathrm{sinc}\;\left(B\;(t-t_{n})\right) \end{array}$$

(11) $$\begin{array}{cc} \; & =\sum_{n\in Z}x\left(t_{n}\right)\;\mathrm{sinc}\;\left(Bt-n\pi \right), \end{array}$$

where $t_{n}=n\;\Delta t$, $\Delta t=\frac{\pi }{B}$, and *B* is the one-sided angular bandwidth, i.e., the highest angular frequency.

The condition BΔt=π, which corresponds to sampling exactly at the Nyquist rate, implies that the time between samples is such that the phase variation in the signal’s fastest harmonic is exactly π. This observation has profound implications and highlights the fundamental importance of phase variation. To clarify, a fundamental parameter in bandlimited function theory is the number of degrees of freedom within an observation interval *T*, given by the time–bandwidth product $\frac{BT}{\pi }$. This quantity, that equals the number of samples required in that interval at Nyquist rate, matches the number of zeros, i.e., the number of π-phase intervals, of the signal’s fastest harmonic over the same interval.

Starting from the above observations, it is possible to discuss the optimal sampling theory from a different point of view based on phase variation in the field.

**Figure 3:**
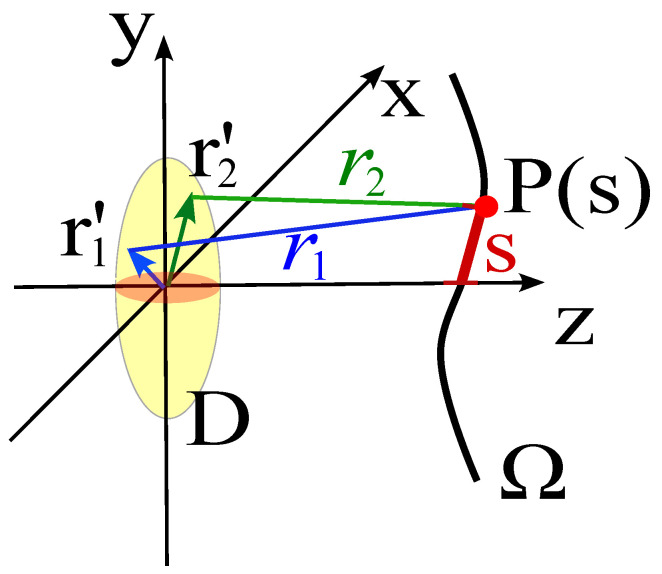
The figure shows a simplified model consisting of two source points belonging to *D*, whose position vectors are $r_{1}^{\prime }$ and $r_{2}^{\prime }$. The field at the point P(s) is restricted to the contribution radiated only by these two sources.

Consider the field radiated by a convex source and observed at a point P(s) on the observation curve, placed at some distance from the source (see Figure 3). In particular, take two elementary radiators having equal amplitude located at $r_{1}^{\prime }$ and $r_{2}^{\prime }$. In a highly simplified model, the field at P(s) can be approximated as

(12) $$\begin{array}{c} E\left(s\right)\;\propto \;\frac{e^{-j\beta r_{1}\left(s\right)}}{r_{1}\left(s\right)}+\frac{e^{-j\beta r_{2}\left(s\right)}}{r_{2}\left(s\right)}, \end{array}$$

where

(13) $$\begin{array}{c} r_{1}\left(s\right)=∥r\left(s\right)-r_{1}^{\prime }∥, \end{array}$$

(14) $$\begin{array}{c} r_{2}\left(s\right)=∥r\left(s\right)-r_{2}^{\prime }∥. \end{array}$$

and the dependence of $r_{1}$ and $r_{2}$ on the abscissa *s* and on the two source points is understood and not explicitly reported.

By factoring the mean and difference distances, and approximate $r_{1}≃r_{2}≃r$ at the denominator (slowly varying amplitude) we have

(15) $$E\left(s\right)\;\propto \;\frac{e^{-j\beta \frac{r_{1}+r_{2}}{2}}}{r}\;2\cos\;\left(\frac{\beta }{2}\;\left(r_{1}-r_{2}\right)\right).$$

The approximation $r_{1}≃r_{2}≃r$ in the denominator is generally valid in the Fraunhofer region. In the present work, however, this expression is used in a somewhat relaxed manner as a pedagogical device to elucidate the physical mechanism underlying optimal sampling.

**Figure 4:**
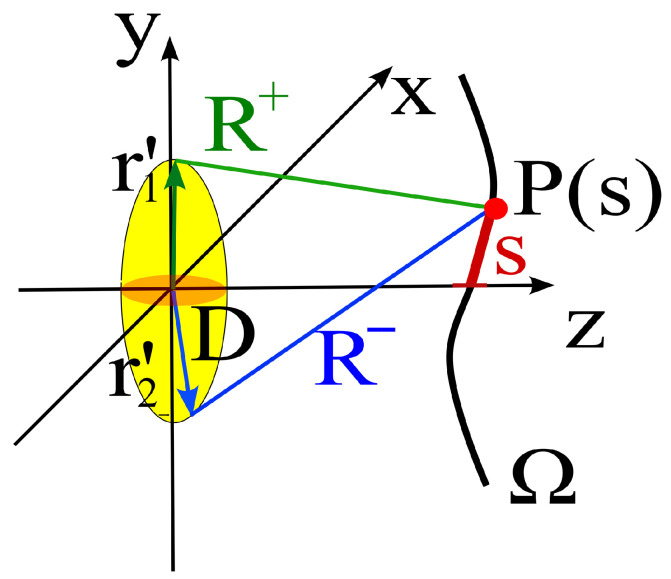
The two distances $R^{+}\left(s\right)$ and $R^{-}\left(s\right)$ associated with the maximum local phase variation around *s*. The rays corresponding to $R^{+}\left(s\right)$ and $R^{-}\left(s\right)$ are tangent to the surface of *D* and intersect it at the extremal points.

From the formula, we can note that among all pairs of source points, the pair that produces the fastest local phase variation near an observation point P(s) is the one for which the distances from P(s) attain, respectively, the local maximum and minimum values; in the following we will denote the two extreme distances by ${\hat{R}}^{+}\left(s\right)$ and ${\hat{R}}^{-}\left(s\right)$ (see Figure 4). Accordingly, for each *s* we identify the two paths that maximize the local spatial frequency of the interference. Note that this requires some a priori information on the position and geometry of the source, that are used to identify the “extremal points” with reference to P(s).

Equation (15) applied to the extremal points identifies two distinct phase terms:

(16) $$\begin{array}{c} \phi^{+}\left(s\right)=\beta \frac{{\hat{R}}^{+}\left(s\right)+{\hat{R}}^{-}\left(s\right)}{2} \end{array}$$

(17) $$\begin{array}{c} \phi^{-}\left(s\right)=\beta \frac{{\hat{R}}^{+}\left(s\right)-{\hat{R}}^{-}\left(s\right)}{2} \end{array}$$

To reduce the phase variation associated with the mean path, we constrain the observation curve to satisfy $\phi^{+}\left(s\right)=\mathrm{const}$, so that the residual (locally dominant) phase variation along *s* is governed solely by $\phi^{-}\left(s\right)$. In other words, constraining $\phi^{+}\left(s\right)=\mathrm{const}$ suppresses the rapidly varying common-mode phase contribution associated with the average propagation path, leaving only the differential phase $\phi^{-}\left(s\right)$, which varies more slowly with *s* and therefore governs the local sampling rate.

As noted earlier, any other pair of source points produces a local phase variation along *s* that is no faster than this. Loosely speaking, these further interferences ’populate’ the bandwidth within *W*, giving a continuous (spatial) spectrum.

Based on the observation that, in the Shannon–Whittaker–Kotelnikov series, the phase advance between adjacent sampling points equals π radians at the highest (spatial) harmonic, we partition the observation curve into segments such that $\phi^{-}\left(s\right)$ changes by π from the beginning to the end of each segment. This “π radians between samples” rule stems from the fact that, after removing the common-mode term $\phi^{+}\left(s\right)$, the field along the curve can be written in the form $E\left(s\right)∼\exp\left(j\phi^{-}\left(s\right)\right)$, where

$$\phi^{-}\left(s\right)=\beta \;\frac{{\hat{R}}^{+}\left(s\right)-{\hat{R}}^{-}\left(s\right)}{2}.$$

This is a complex baseband representation, so the Nyquist condition corresponds to a phase increment of π between consecutive samples. Moreover, the expression of ξ(s) in Equation (9) makes this connection explicit, since

$$\phi^{-}\left(s\right)=\beta \;a\;\xi \left(s\right)\;\Rightarrow \;\frac{d\phi^{-}\left(s\right)}{ds}=\beta \;a\;\frac{d\xi (s)}{ds}.$$

Hence, the local sampling step along *s* is determined by the Nyquist relation

$$\Delta s≃\frac{\pi }{\beta a}\;\frac{1}{\left|d\xi (s)/ds\right|},$$

which directly links the π-spacing criterion to the normalized differential coordinate defined in Equation (9). This method yields a sampling density consistent with the Nyquist bound and ensures that, in the worst case, the phase excursion between neighboring samples does not exceed π radians.

In essence, the two functions $\phi^{+}\left(s\right)$ and $\phi^{-}\left(s\right)$ identify the natural geometric quantities underpinning field sampling. At a fundamental level, this geometry is fixed by the time propagation of the electromagnetic field from the sources to the observation point, which in the harmonic domain corresponds to phase. Indeed, in the geometry shown in Figure 4, the Ψ(s) curves correspond to $\phi^{+}\left(s\right)$, and the ξ(s) curves correspond to $\phi^{-}\left(s\right)$ when the effective bandwidth *W* is normalized to one. Note that, in the optimal sampling scheme, if the observation curve does not match the natural spatial sampling geometry (i.e., is not a $\phi^{+}\left(s\right)=\mathrm{const}$ curve), the value of $\phi^{+}\left(s\right)$ is subtracted from the field to obtain the reduced field (see Equation (5)). In [3], it is rigorously demonstrated that this is the best choice to compensate for the extra mean phase shift (i.e., the extra time delay in a time-domain interpretation) that arises on curves where $\phi^{+}\left(s\right)$ is not constant, while Appendix A of this paper provides an intuitive explanation of this result.

**Figure 5:**
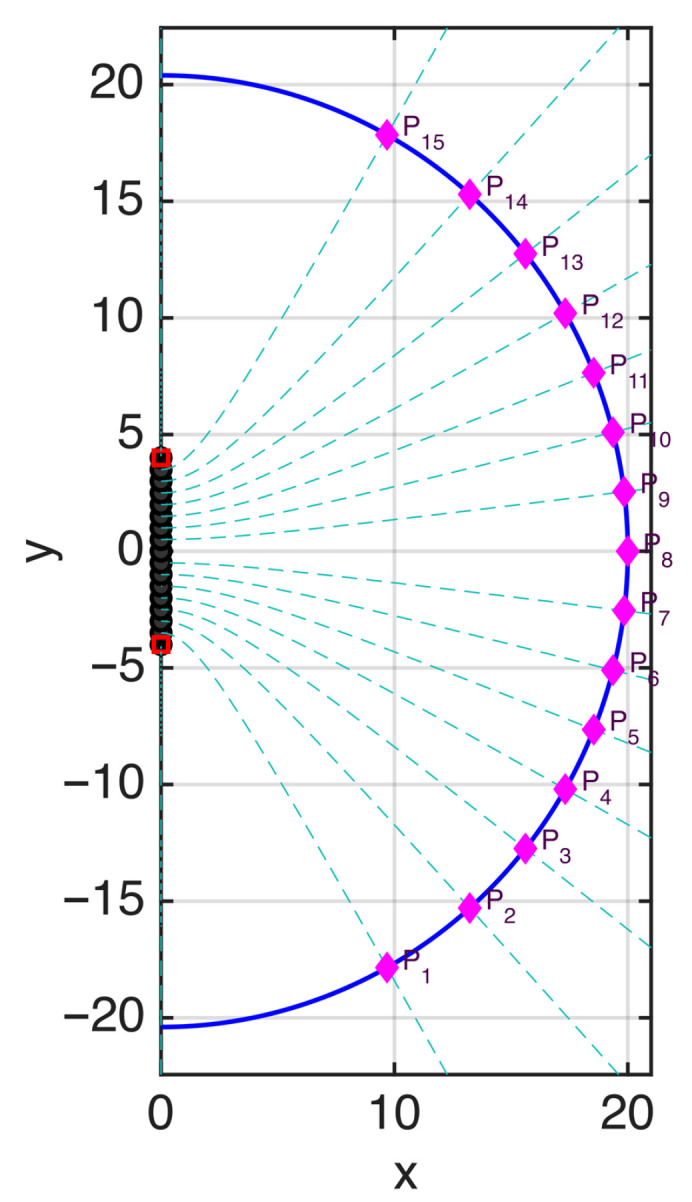
Linear source and observation curve with $\phi^{+}\left(s\right)=\mathrm{const}$. Point $P_{8}$ corresponds to s=0. The points where $\phi^{-}\left(s\right)=n\pi$ are shown in pink. The $\xi_{n}$ curves of the optimal sampling theory, computed for a bandwidth w=W (with *W* denoting the effective bandwidth in the optimal sampling theory), are plotted as dashed lines. The sampling points of the optimal sampling theory are the intersections between the $\xi_{n}$ curves and the observation curve, and coincide with the abscissas $s_{n}$ such that $\phi^{-}\left(s_{n}\right)=n\pi$.

To better clarify the connection between the rigorous theory and the heuristic approach, let us consider a linear source of length ℓ=2a=8λ (see Figure 5) and the curve $\phi^{+}\left(s\right)$ in the x>0 half-space (i.e., a semi-elliptical observation curve) intersecting the *x*-axis at x=20λ. The points $s_{n}$ where $\phi^{-}\left(s_{n}\right)=n\pi$, which define the heuristic sampling positions, are plotted in the same figure.

For comparison, the curves $\xi_{n}=\mathrm{cost}.$, adopted in the optimal sampling theory, are also plotted as dashed lines. The figure confirms that the sampling points obtained from this partition of the observation curve coincide with the sampling positions of the optimal strategy, given by the intersection points between the $\xi_{n}$ curves as defined in [3] and the observation curve.

**Figure 6:**
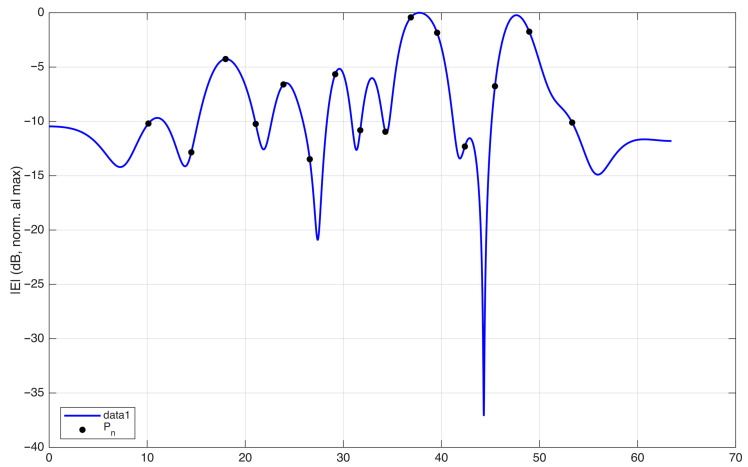
Amplitude of the field (dB) along the observation curve for a random-amplitude, random-phase source. The sampling points identified in Figure 5 are shown as black circles. In optimal sampling theory, the same points are obtained by considering a spatial bandwidth w=W, i.e., without oversampling.

**Figure 7:**
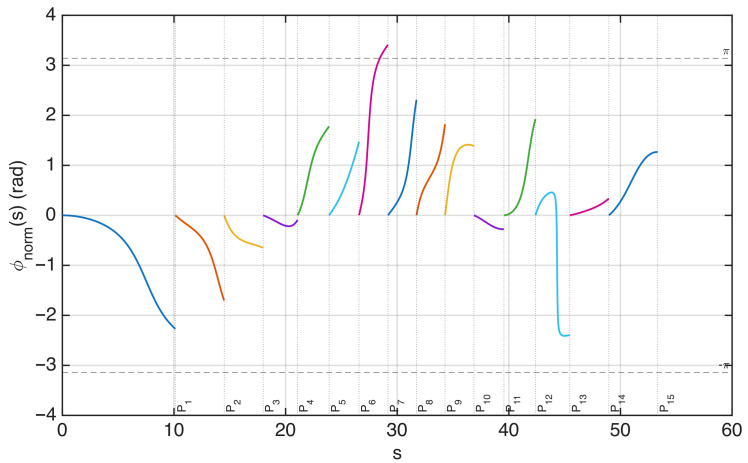
Phase of the field along the observation curve, normalized to zero at each sampling point identified in Figure 5. The plot shows that, in the interval between the two sampling points $P_{6}$ and $P_{7}$, the phase variation exceeds π. The same representation using ξ(s) instead of *s* would change the spacing between the samples, making all intervals equal in length.

In Figure 6, the field on the observation curve and the sampling points are plotted, considering an antenna excited by random-amplitude, random-phase currents. The corresponding phase along the observation curve is plotted in Figure 7. In this figure, the phase of the field is normalized to 0 radians at each sampling position to visualize the phase variation in the interval between two adjacent points. The normalized phase is denoted by $\phi_{norm}$ in the figure. The same representation using ξ instead of *s* would change the spacing between the samples, making all intervals equal in length.

The plot shows that the phase variation remains within π in almost all intervals, except one. This indicates that the sampling points $s_{n}$ defined by $\phi^{-}\left(s_{n}\right)=n\pi$ are not sufficient to represent the field without aliasing.

This observation is consistent with the oversampling prescribed by the optimal sampling theory. In practice, choosing the criterion $\phi^{-}\left(s_{n}\right)=n\pi$ is equivalent to applying the optimal sampling strategy with a nominal bandwidth *w* equal to the effective bandwidth *W*. In the rigorous theory, *W* plays the role of a critical value: the approximation error remains large for w<W, whereas it becomes small for w>W. To obtain a sufficiently small band limitation error, the theory requires a bandwidth w=χW with χ>1, i.e., an oversampling with respect to a signal of bandwidth *W*. The interpolation error decreases rapidly as χ increases [3].

**Figure 8:**
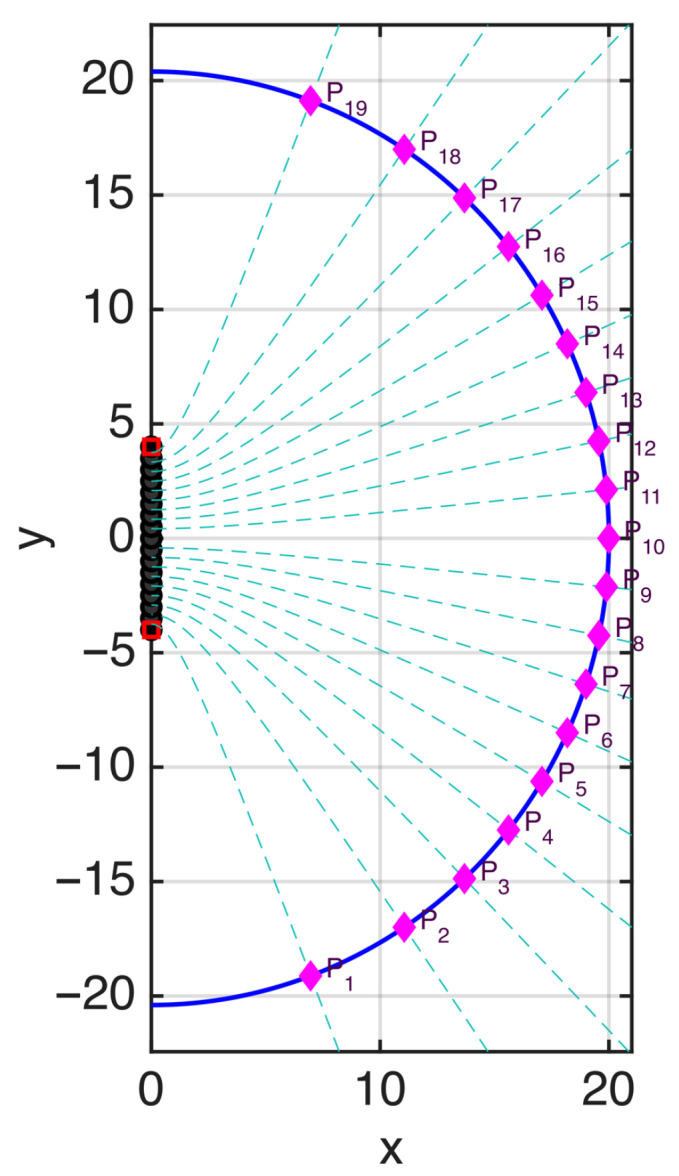
The points at positions $s_{n}$ satisfying $\phi^{-}\left(s_{n}\right)=n\pi /\chi$ are plotted as pink dots. The $\xi_{n}$ curves of optimal sampling theory, obtained by considering a spatial bandwidth w=χW with χ=1.2, are plotted as dashed lines. The sampling points of the optimal theory coincide with the $s_{n}$ points.

**Figure 9:**
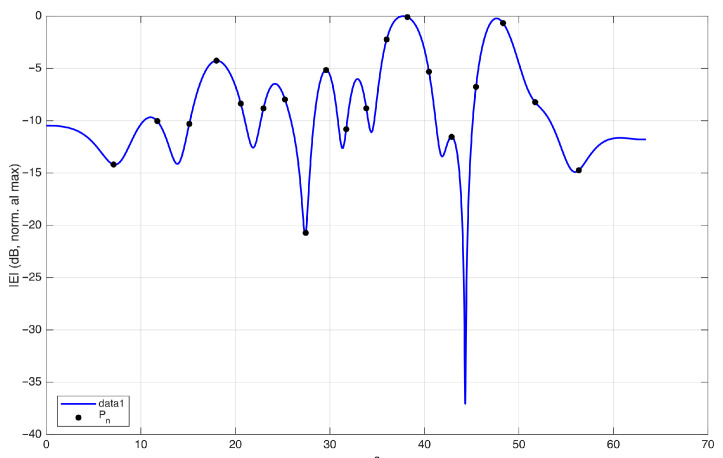
Amplitude of the field (dB) on the observation curve for a random-amplitude, random-phase source. The sampling points are plotted as black circles. In optimal sampling theory, these points are obtained by considering a spatial bandwidth w=χW with an oversampling factor χ=1.2.

**Figure 10:**
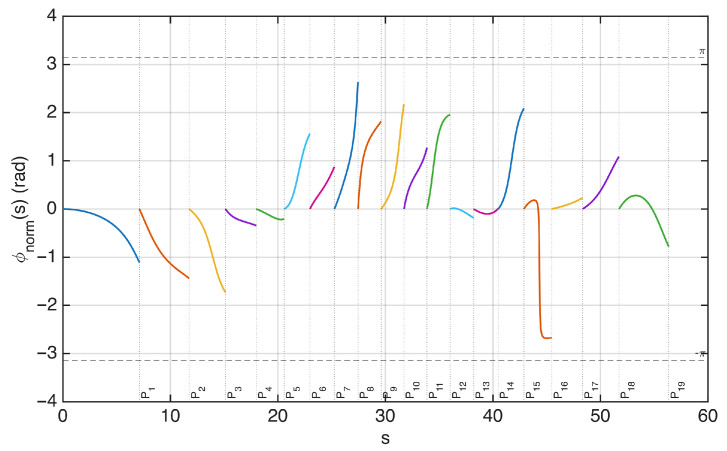
Phase of the field along the observation curve, normalized to zero at each sampling point satisfying $\phi^{-}\left(s\right)=n\pi /\chi$ with χ=1.2. In all intervals between consecutive sampling points, the phase variation remains within π. The same representation using ξ(s) instead of *s* would change the spacing between the samples, making all intervals equal in length.

In the heuristic approach, this is equivalent to placing the samples at $s_{n}$ defined by $\phi^{-}\left(s_{n}\right)=n\pi /\chi$. As an example, in Figure 8 a value χ=1.2 is used to select the sampling points along the curve. The resulting sampling positions are shown in Figure 9. Due to the shorter spacing between samples, the phase variation in the field now remains within π in all intervals, allowing a field reconstruction with negligible aliasing error (see Figure 10).

As a last observation, this physics-based approach is, at least in principle, suitable for extension to 2D observation surfaces. This would be of practical relevance, since sampling on surface observation domains is cumbersome and, to the best of the author’s knowledge, not yet completely solved. A simple strategy is to leverage the results obtained on curves by considering the coordinate curves on the surface [1,3]. This approach matches the probe movement in near-field antenna measurement systems. However, the resulting sampling grid tends to be redundant. In particular, sampling grids derived from optimal sampling along curves exhibit a redundancy factor of 4/π with respect to the NDF [12], i.e., they require about 1.27 times more samples than the theoretical minimum, to which one must add the oversampling required to control the band limitation error. This can be easily illustrated in the case of planar sources, for which the visible spectral region corresponds to a disk of area $\pi \beta^{2}$. In the optimal sampling framework, however, the spectral support is a square of side 2β, i.e., an area $4\beta^{2}$. The resulting spectral area is therefore larger by a factor 4/π, and this excess is associated with non-propagating contributions.

A possible strategy to extend the 1D approach, i.e., sampling based on local phase variation, to a field observed on a 2D surface is to consider the phase gradient. The 2D analogue of the 1D adaptive interval is an unstructured sampling mesh in which the placement of samples (i.e., the mesh vertices) is governed directly by the local phase variation, keeping the phase variation within π along each mesh edge. It must be pointed out that reconstructing a signal from an unstructured grid is numerically more complex than the standard 2D Shannon–Whittaker–Kotelnikov expansion.

A more direct extension of rigorous one-dimensional (1D) sampling theory to two-dimensional (2D) surfaces involves identifying a closed-form warping transformation. A warping transformation is a nonlinear change in variables that converts a non-uniformly bandlimited field into a uniformly bandlimited one, thereby enabling classical Shannon sampling on a uniform grid [13]. Consequently, the transformation adopted for optimal sampling along curves can be interpreted as a warping transformation derived from the physical characteristics of the function being sampled, effectively unifying physics and sampling theory. Identifying a closed-form warping transformation of the electromagnetic field over 2D surfaces remains an active area of research [14,15].

## 5. Conclusions

In this paper, an intuitive approach to the optimal sampling method proposed in [1,3] has been presented. This perspective offers a simple physical interpretation of the underlying principles of the theory.

In particular, the intuitive approach reveals the role of interference phenomena and suggests a natural connection between sampling theory and the theory of electromagnetic field spectral coherence. This connection has been demonstrated in [16], and reflects more fundamental properties of the electromagnetic field that link electromagnetics and information. In this sense, the concepts discussed in this work extend well beyond near-field sampling applications, and are relevant to the emerging discipline of ESIT (Electromagnetics, Signal and Information Theory) [17]. A video following an intuitive approach to the relationship between sampling and ESIT is also available [18].

Before concluding this paper, it should be emphasized that optimal field sampling rests on a rigorous mathematical framework grounded in approximation theory and functional analysis. Physical intuition is helpful when setting sail toward unfamiliar waters, but without mathematical rigor—the set of rules that allows us to navigate safely—it risks leading to fragile reasoning and costly errors. The approach outlined in this paper is not intended to supplant the rigorous theory of optimal sampling. Rather, it serves as an accessible introduction, designed to illuminate the fundamental principles and encourage a more in-depth study of the complete, rigorous, and mathematically elegant framework.

In other words, it is merely a brief blurb meant to encourage the reader to read the entire book.

## Figures and Tables

**Figure A1:**
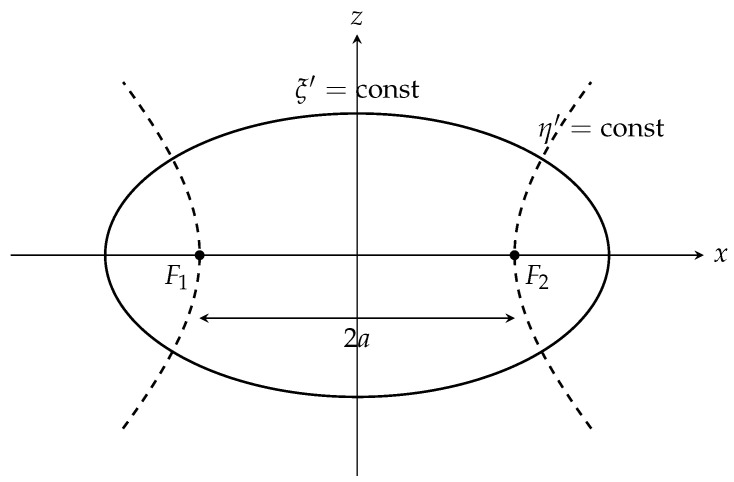
Two-dimensional section at $\phi^{\prime }=\mathrm{const}$ of the prolate spheroidal coordinate system $\left(\xi^{\prime },\eta^{\prime },\phi^{\prime }\right)$ with focal distance 2a. The curves $\xi^{\prime }=\mathrm{const}$ correspond to prolate spheroids with foci at the endpoints of the line source, while the curves $\eta^{\prime }=\mathrm{const}$ correspond to two–sheeted hyperboloids. By revolving this section around the *x*-axis, the full three-dimensional prolate spheroidal surfaces are obtained.

## Data Availability

The original contributions presented in this study are included in the article. Further inquiries can be directed to the corresponding author.

## Appendix A. On the Minimum–Mean Phase–Variation Property of *ϕ*^+^ (s)

This appendix provides an intuitive rationale for the minimum–mean phase–variation property of the curves $\phi^{+}\left(s\right)=\mathrm{const}$, clarifying the role of prolate spheroidal coordinates and the notation used. In the main text it has been shown that, in the geometry of Figure 4, the functions Ψ(s) and ξ(s) correspond, for unit effective bandwidth *W*, to the phase functions $\phi^{+}\left(s\right)$ and $\phi^{-}\left(s\right)$, respectively. Thus, $\phi^{+}\left(s\right)=\mathrm{const}$ describes the curves on which the dominant propagation phase (or, in the time domain, the dominant propagation delay) is approximately constant, and it is therefore natural to expect these curves to minimize the mean phase variation.

As a preliminary step, recall that in prolate spheroidal coordinates $\left(\xi^{\prime },\eta^{\prime },\phi^{\prime }\right)$ the surfaces $\xi^{\prime }=\mathrm{const}$ are prolate spheroids with foci at the endpoints of the focal segment (see Figure A1); in the limit $\xi^{\prime }\to 1$ the spheroid degenerates to the focal line segment. Thus, a finite line source of length 2a can be viewed as the degenerate limit of $\xi^{\prime }=\mathrm{const}$ spheroidal surfaces.

Consider now the three-dimensional geometry. For a uniform, azimuthally symmetric line current of length 2a, solutions of the scalar Helmholtz equation can be expressed in terms of prolate spheroidal wave functions [19]. The electrical size is characterized by the spheroidal parameter

$$c^{\prime }=\beta a,$$

where β is the wavenumber and *a* is half the length of the line source. For an azimuthally symmetric configuration (azimuthal index m=0 in the spheroidal functions), the field can be expanded as

(A1) $$A\left(\xi^{\prime },\eta^{\prime }\right)\;=\;\sum_{n=0}^{\infty }C_{n}\;R_{0n}^{\left(4\right)}\left(c^{\prime },\xi^{\prime }\right)\;S_{0n}\left(c^{\prime },\eta^{\prime }\right)\;.$$

In (A1),

- $A(\xi^{\prime },\eta^{\prime })$ is the scalar field (e.g., a component of the electric field) expressed in prolate spheroidal coordinates;
- $C_{n}$ are complex modal coefficients determined by the source distribution;
- $R_{0n}^{\left(4\right)}\left(c^{\prime },\xi^{\prime }\right)$ are the radial spheroidal functions of the fourth kind, representing outgoing-wave solutions;
- $S_{0n}\left(c^{\prime },\eta^{\prime }\right)$ are the angular spheroidal functions;
- $c^{\prime }=\beta a$ is the spheroidal parameter, proportional to the electrical size of the source.

For real $c^{\prime }$ and m=0, the functions $S_{0n}\left(c^{\prime },\eta^{\prime }\right)$ can be taken real, so that they weight the modal amplitudes, whereas the complex-valued $R_{0n}^{\left(4\right)}\left(c^{\prime },\xi^{\prime }\right)$ carry the dominant propagation phase.

Fix a surface $\xi^{\prime }=\xi_{0}^{\prime }$. On this surface, the functions $S_{0n}\left(c^{\prime },\eta^{\prime }\right)$ act as real weighting factors on the phasors $C_{n}R_{0n}^{\left(4\right)}\left(c^{\prime },\xi_{0}^{\prime }\right)$. The superposition produces some phase variation with $\eta^{\prime }$ (including π-phase flips at the zeros of $S_{0n}$), but this hmodal interference is typically much smaller than the rapid radial phase progression along $\xi^{\prime }$, governed by $R_{0n}^{\left(4\right)}\left(c^{\prime },\xi^{\prime }\right)$. In particular, for electrically large sources ($c^{\prime }\gg 1$), the phase of $R_{0n}^{\left(4\right)}\left(c^{\prime },\xi^{\prime }\right)$ varies on the order of βa along the radial direction, whereas the phase changes induced by the angular factors $S_{0n}\left(c^{\prime },\eta^{\prime }\right)$ remain bounded to values of order unity (at most a few radians) over the full angular range [19,20]. Hence, the radial phase gradient dominates the overall variation, and along a surface $\xi^{\prime }=\mathrm{const}$ the phase exhibits a minimum mean variation when compared with the much faster variations along $\xi^{\prime }$.

From a geometric viewpoint, the surfaces $\xi^{\prime }=\mathrm{const}$ are level sets of the sum of the distances from the two foci. In the high-frequency regime, this sum of distances is precisely the quantity that controls the dominant propagation phase of the field, i.e., it plays the role of $\phi^{+}$. Thus, in this sense, $\xi^{\prime }=\mathrm{const}$ surfaces are the natural “equal–$\phi^{+}$” surfaces for a line source.

Returning to the two-dimensional setting considered in the paper, the curve $\phi^{+}\left(s\right)=\mathrm{const}$ is an ellipse with foci at the endpoints of the line source, i.e., a two-dimensional section of a $\xi^{\prime }=\mathrm{const}$ spheroidal surface. Consequently, it is the curve of (approximately) least phase variation for a line source in the (x,y)-plane. This explains why $\phi^{+}\left(s\right)$ is the natural phase to be removed in the optimal sampling scheme: subtracting $\phi^{+}\left(s\right)$ compensates for the dominant propagation delay, leaving a reduced field whose residual phase variation is as small as possible along the observation curve.
